# Induced Genetic Variation in Crop Plants by Random or Targeted Mutagenesis: Convergence and Differences

**DOI:** 10.3389/fpls.2019.01468

**Published:** 2019-11-14

**Authors:** Inger B. Holme, Per L. Gregersen, Henrik Brinch-Pedersen

**Affiliations:** Department of Molecular Biology and Genetics, Research Center Flakkebjerg, Aarhus University, Slagelse, Denmark

**Keywords:** conventional mutagenesis, EU legislation, New Breeding Techniques (NBT), NBT mutagenesis, precision breeding, off-target mutations

## Abstract

New Breeding Techniques (NBTs) include several new technologies for introduction of new variation into crop plants for plant breeding, in particular the methods that aim to make targeted mutagenesis at specific sites in the plant genome (NBT mutagenesis). However, following that the French highest legislative body for administrative justice, the Conseil d’État, has sought advice from The Court of Justice of the European Union (CJEU) in interpreting the scope of the genetically modified organisms (GMO) Directive, CJEU in a decision from 2018, stated that organisms modified by these new techniques are not exempted from the current EU GMO legislation. The decision was based in a context of conventional plant breeding using mutagenesis of crop plants by physical or chemical treatments. These plants are explicitly exempted from the EU GMO legislation, based on the long-termed use of mutagenesis. Following its decision, the EU Court considers that the NBTs operate “at a rate out of all proportion to those resulting from the application of conventional methods of mutagenesis.” In this paper, we argue that in fact this is not the case anymore; instead, a convergence has taken place between conventional mutagenesis and NBTs, in particular due to the possibilities of TILLING methods that allow the fast detection of mutations in any gene of a genome. Thus, by both strategies mutations in any gene across the genome can be obtained at a rather high speed. However, the differences between the strategies are 1) the precision of the exact site of mutation in a target gene, and 2) the number of off-target mutations affecting other genes than the target gene. Both aspects favour the NBT methods, which provide more precision and fewer off-target mutations. This is in stark contrast to the different status of the two technologies with respect to EU GMO legislation. In the future, this situation is not sustainable for the European plant breeding industry, since it is expected that restrictions on the use of NBTs will be weaker outside Europe. This calls for reconsiderations of the EU legislation of plants generated *via* NBT mutagenesis.

## Introduction

Plant breeding is a discipline for targeted and continuous development of new plant varieties. It utilizes the genetic variation between individuals within a plant species and combines the desired properties into new and improved varieties. Plant breeding is dependent on genetic variation, and new variation is fundamentally important for introduction of new traits in breeding programs. However, in cases where a specific genetic trait is not immediately available to be crossed into breeding materials, the genetic variation in a crop species can be expanded by other means. For decades this has been achieved by, e.g., chemical or physical treatments, translocation breeding, synthetic hexaploids, etc; techniques that involve comprehensive changes of the plant’s genome. Due to its long safety record, organisms obtained by physical and chemical mutagenesis are exempt from the provisions of the GMO legislation in the EU. Nevertheless, the methods incite hundreds or even thousands of random mutations with unknown effects and consequences.

New Breeding Techniques (NBT) include several new technologies for introduction of variation into crop plants. NBT comprises a number of technologies that have emerged since the current Directive 2001/18/EC on GM plants was implemented. At the request of the member states, the European Commission set up a working group in 2007 to assess whether or not a number of new breeding techniques should fall within the scope of GMO legislation. The working group prepared a list of seven new plant breeding techniques: zinc finger nuclease (ZFN) technology, oligonucleotide-directed mutagenesis (ODM), cisgenesis and intragenesis, grafting on GM-rootstock, RNA-dependent DNAmethylation, agro-infiltration “sensu stricto,” and reverse breeding. The ZFN technique is a site-directed nuclease (SDN) tool that can be designed to produce a mutation at a predetermined position in the plant genome. Since 2007, a number of new SDN tools have emerged, such as the TALEN and CRISPR/Cas techniques, of which, in particular, the latter is now widely used. It is beyond the scope of this paper to describe all the different NBTs in detail. Here we will focus on the two techniques involved in the generation of mutations at pre-determined sites in a plant genome, i.e., ODM and especially the SDN-tools. We will refer to these as NBT mutations and use the term precision breeding to describe the use of NBT mutations in plant breeding. The other NBTs are described in detail in (Lusser et al., 2012) and (Schaart et al., 2016). Common to almost all these techniques are, however, that the final plants, which are exposed to the open environment, are without foreign DNA as the vector constructs are either never integrated into the plant genome or are out-segregated in the next generation. Exceptions are cisgenesis/intragenesis, and the use of the SDN-tools to insert longer DNA fragments into pre-selected sites in the plant genome. Both of these techniques require that the transferred DNA is permanently integrated into the plant genome.

Early after their emergence, the SDN technologies were adopted to improve mutations already available from traditional mutagenesis. For example, in order to improve the quality of soy oil and avoid non-ideal mutations induced by traditional mutation, two target genes FAD2-1A and FAD2-1B were simultaneously mutated using TALENs (Haun et al., 2014). Functional mutations down to the deletion of two nucleotides were identified. In contrast, traditional induced mutations of FAD2-1A by x-ray are up to 164-kb deletions that may remove other desirable genes in addition to FAD2-1A (Bolon et al., 2011). In another early example, fragrant rice was generated by a SDN directed toward 1-bp deletion in the gene encoding betaine aldehyde dehydrogenase (BADH2). Traditionally induced mutations in the gene mutation are up to 803-bp deletions plus a range of unknown side mutations (Shan et al., 2015). Placed in the context of traditional mutagenesis methodologies in crops, the examples demonstrate how SDN technology improves precision and reduces the extent of mutations in a crop where a specific trait is pursued.

Only a few crops have been improved through the use of ODM, whereas the SDN tools are widely used. Without doubt, NBT mutations represents a significant progress for the breeding of crops for a challenging future. Speed and precision are often mentioned as key beneficial properties of the NBT mutations. However, on July 25, 2018, The Court of Justice of the European Union (CJEU) ruled that organisms obtained by these new mutagenesis technologies are not exempted from the current EU GMO legislation. (Court-of-Justice-of-the-European-Union, 2018) In order for precision bred crops to be exempted, the GMO Directive needs to be revised to reflect scientific progress in biotechnology. In the discussion of this, parallels must be drawn to the use of conventional mutation breeding as a way of inducing genetic variation in breeding material. Hence, it is worthwhile as a first step to look more closely into this approach; how it has been used and developed in plant breeding; and how it compares to the new targeted genome editing techniques, in particular the CRISPR/Cas9-based techniques. What are the differences and are there significant convergence between the old methods exempted from GM legislation and NBT mutations. In order to place NBT mutations in the context of today’s breeding, we will in the current paper uncover major similarities and differences between NBT mutations and mutations obtained by conventional mutagenesis with respect to precision and off-target mutations.

### Conventional Mutation Breeding in the Context of New Breeding Technologies

Mutation breeding has been used by plant breeders world-wide since the discovery in the 1920s that heritable mutations could be induced in plants by means of irradiation or chemical treatments (Stadler, 1928). The expectations to this method for improvements of crop varieties were big in the 1950s to 1960s, and indeed a considerable number of varieties was released, e.g. from Scandinavian barley breeding (Lundqvist, 2014). The mutated genes from these old mutant varieties are still part of the gene pool used for modern barley breeding. Since the 1980s, the interest among plant breeders in using mutation breeding has declined, probably due to expectations to the new genetic modification technologies (GM traits), but also due to difficulties in dealing with the load of accompanying bad mutations in selected lines, which hampered development of high-yielding varieties based on mutations (Mba, 2013). Nevertheless, even with the harsh treatments of plants in the mutation breeding process aimed to induce genetic modifications, the plants coming out of it were explicitly exempted from the EU GMO legislation on GM crops, implemented almost 30 years ago (Directive 90/220/EEC and Directive 2001/18/EC), due to their long safety record.

Irradiation and treatment with chemical mutagens are the two major methods used to induce mutations in plants (Leitao, 2011; Mba et al., 2011). X-rays and gamma radiation cause a mixture of bigger chromosome deletions and point mutations, i.e., single base substitutions or deletions, whereas the most commonly used chemical mutagens (e.g. NaN_3_, EMS, MNU) almost exclusively cause single base substitutions (transitive, i.e. from G/C to A/T; see **Table 1**). The advantage of the chemical mutagens is that they can be used to prepare mutant populations with high densities of mutations, making it easier to screen for specific mutations in a population (Szarejko et al., 2017).

**Table 1:** Mutagens commonly used in mutation breeding and in generation of TILLING mutant populations (Leitao, 2011; Mba et al., 2011; Sikora et al., 2011).

Category	Mutagen	Mutation type	Genotype
Physical treatment	X-rays	Dependent of dose: mixture of gene mutations and chromosomal mutations/rearrangements	Point mutations.
	Gamma irradiation		Deletions/inversions of varying sizes. Translocations.
Chemical treatment	Alkylating mutagens: • MNU • EMS • ENU	Gene mutations	Alkylated base mispairing, typically leading to G/C→A/T transitions
			Few InDels
	NaN_3_	Gene mutations	Both G/C→A/T og A/T→G/C transitions
			Few InDels

EMS, ethyl methanesulphonate; MNU, N-methyl-N-nitrosourea; ENU, 1-ethyl-1-nitrosourea; NaN_3_, natriumazide, InDel, insertion/deletion.

By the turn of the century, a big change with respect to utilization of mutants took place following development of efficient TILLING (Targeting Induced Local Lesions in Genomes) techniques (Mccallum et al., 2000). Previously, mutation breeding was exclusively based on forward genetics, i.e. on phenotype screening for favorable traits in mutant populations. TILLING made reverse genetics approaches applicable, since this technique is aimed at the detection of mutations in specific, known genes. The use of TILLING has accompanied the general development of molecular insight into the genetic base for crop traits and development of efficient new generation DNA sequencing techniques. In principle, this now makes it possible to find mutations in any pre-selected gene across the genome of crop plants, if the DNA sequence of the gene is known and if a suitable mutant population is available (Jankowicz-Cieslak et al., 2017).

For the major European crops, barley and wheat, good TILLING population resources are already existing (Krasileva et al., 2017; Szurman-Zubrzycka et al., 2018), and for most seed propagated species TILLING populations can in principle be generated, if not available. In addition, new generation sequencing techniques (Burkart-Waco et al., 2017; Krasileva et al., 2017) and efficient methods to detect DNA heteroduplexes (Szurman-Zubrzycka et al., 2017) have made it easier than previously to screen the populations for mutations in selected target genes. Since the alkylating chemical mutagens mainly cause transitions in the chromosomal DNA, specific mutations can to a certain degree be predicted and searched for during screening of a mutant population. Hence, mutagenesis is not just a random tool to discover mutations, but can be partially directed. Furthermore, DNA marker-assisted backcrossing can make the transfer of mutations into elite varieties much more efficient than previously (Hasan et al., 2015).

Overall, the development of TILLING, stable mutant populations, and efficient backcrossing in principle now makes the acquisition of mutations in specific genes very efficient and fast for seed propagated crop species. This contrasts with the statement that were made by the CJEU in its 2018 decision on NBTs, namely that the new breeding “techniques make it possible to produce genetically modified varieties at a rate out of all proportion to those resulting from the application of conventional methods of mutagenesis” (https://curia.europa.eu/jcms/upload/docs/application/pdf/2018-07/cp180111en.pdf). The situation now is that mutations can be acquired at a high rate as well with the conventional techniques. This sets a new scene for comparing the new precision breeding techniques, in particular those based on CRISPR/Cas9, with the conventional mutation breeding techniques. Hence, with this development there has been a convergence between the new breeding techniques and the efficient use of TILLING principles in reverse genetics approaches. This applies to the general ability to achieve mutations in any gene of interest, but also to the speed of the process.

Despite the convergence between targeted genome editing and TILLING approaches there are still two main differences, which count in favor of NBT genome editing techniques: 1) precision – genome editing has only few constraints with respect to selection of the exact site of mutation in a gene, whereas TILLING is based on random mutations across the entire genome; 2) off-target mutations – genome editing can result in a few off-target mutations, whereas a high load of off-target mutations is an intrinsic property of conventional mutagenesis that has to be dealt with in plant breeding *via* extensive backcrossing strategies.

### Mutations Induced by the NBT Mutation Tools

As previously mentioned, the most widely used NBT mutation tool in plants is CRISPR/Cas9. This is mainly because it is highly efficient and easy to design and because it is possible by multiplexing to make more than one targeted mutation at a time (e.g. Bortesi and Fisher, 2015). However, ZNF, TALENs, and ODM are also currently used. ZFN was developed in 2003 (Bibikova et al., 2003), TALENs in 2011 (Bogdanove and Voytas, 2011) and CRISPR/Cas9 in 2012 (Jinek et al., 2012), so the SDN-tools are less than 20 years old. ODM, on the other hand, is a tool that has been used for a long time across mammalian, microbial, and plant systems to induce mutations at a specific site in the genome and ODM started to be successfully used in plants around 20 years ago (Breyer et al., 2009). Thus, NBT mutagenesis is almost 80 years younger than conventional mutagenesis.

#### Mechanism Behind the NBT Mutation Tools

The mechanism behind the precision of the ODM and SDN mutation tools are very different. ODM makes use of oligonucleotides (between 20 to 100 nucleotides in length) designed to be identical to a corresponding sequence in the plant genome except for one or a few altered nucleotides corresponding to the intended mutation (Breyer et al., 2009). The oligonucleotides bind to the complementary DNA sequence in the genome, thereby generating mismatches, which are repaired by the DNA repair system of the cell. As a result, a desired change is achieved at a specific site in the genome. The efficiency of this technique is, however, very low, and site-directed nucleases, especially the CRISPR/Cas tool, are therefore currently the preferred tool for creating NBT mutations.

SDNs are tools that can be designed to recognize and cleave at specific sites within a genome and thereby create a double strand break (DSB) at the targeted site. Mutations can then, prone to some error rate, be generated in the subsequent repair of the DSB performed by the cell’s own DNA repair systems (Voytas, 2013). The DSB enables the creation of different types of mutations by harnessing the DSB repair pathways of the cell. The different types of mutations obtained by the two primary repair pathways, non-homologous end-joining and homologous recombination, are often referred to as SDN1 and SDN2, respectively.

The most commonly used repair pathway of DSBs is non-homologous end-joining (NHEJ) in which the broken DNA strands are just simply rejoined. When the rejoining is imprecise, deletions or insertions are introduced at the site of the DSB. If the SDN-tool is designed to make a DSB in a gene sequence, imprecise rejoining can inactivate the gene by changing the amino acid sequence reading frame.

The other main repair system of DSBs in cells is homologous recombination (HR). This repair requires the presence of a DNA fragment with sequence homology to either site of the DSB which can be used as a template for the HR repair. Specific nucleotide changes in the genomic sequence at the site of the DSB can then be achieved through HR by designing a DNA repair template with homologous sequences to either side of the DSB, but with the desired nucleotide changes at the site of the DSB. When the DNA repair template is delivered to the cell along with the SDN-tool, the template can be used for HR repair of the DSB and the template with the nucleotide changes will be incorporated into the chromosome, thereby specifically replacing one or a few nucleotides to other desired nucleotides. In this way, the genetic code of an amino acid can be changed to the code of another amino acid. Replacing a single amino acid in an enzyme often can alter the activity or specificity of the enzyme. Therefore, if already known which amino acid that has to be replace to achieve an improvement, SDN2 can be used to induce the corresponding specific nucleotide change.

Changing a single or a few nucleotides using SDN2 is, however, difficult as the delivery of the SDN-tool to the cell must be coordinated with the delivery of the DNA repair template. Thus, new approaches to overcome this hurdle are currently developed. Two base editing systems based on the CRISPR/Cas9 tool have recently been developed which can alter a particular nucleotide in a DNA sequence without the use of a DNA repair template (reviewed by (Shan and Voytas, 2018). One system can change cytosine (C·G) to thymine (T·A) (Zong et al., 2017) and the other adenine (A·T) to guanine (G·C) (Li et al., 2018). These systems have been shown to work effectively in important crop plants, such as tomato, canola, corn, rice, and wheat (Shan and Voytas, 2018).

#### Constrains of NBT Mutations With Respect to Target Site and Traceability

As compared to conventional mutagenesis, there are only few constraints when selecting the exact site for NBT mutations. Although only few there are some constrains, in particular for the CRISPR/Cas system. It consists of a Cas nuclease inducing the DSB and a chimeric RNA (gRNA) where the first 20 nucleotides (the guide sequence) can be made complementary to a 20-nucleotide genomic sequence located where the mutation is intended (Jinek et al., 2012). The gRNA strand and the Cas nuclease forms the RNP complex, and together they will find and bind to the complementary nucleotides in the genome. Here the Cas nuclease will cleave the DNA double strand but only if a protospacer adjacent motif (PAM sequence) is present just in front of the 20 bp targeted DNA sequence in the genomic sequence. The most commonly used Cas nuclease is spCas9 which originates from *Streptococcus pyogenes*. The PAM sequence for spCas9 is NGG. Although the NGG sequence is abundant in plant genomes, the requirement for a particular PAM sequence represents a restriction on where in the genome a DSB can be induced. However, to overcome this, new engineered spCas9 nucleases or CRISPR/Cas systems identified in other bacteria requiring other PAM sequences are now available and can be used in the absence of a wild type spCas9 PAM sequence at sites where a DSB is desired (Kleinstiver et al., 2015; Ran et al., 2015; Kleinstiver et al., 2016b; Kim et al., 2017; Amrani et al., 2018).

A key difference between conventional mutation breeding and NBT mutations is that the NBT mutation tools have to be delivered into the cells. For ODM, the oligonucleotides are transiently delivered to the cell and are degraded in the cells after induction of the specific mutation. The SDN-tools can be delivered to the plant cells as DNA constructs either using stable or transient transformation techniques. Although mutated primary transformants generated by stable transformation contains the SDN DNA-construct there is most frequently no linkage between the site of insertion of the construct and the site of the mutation. Mutants without the DNA construct can therefore be selected in the subsequent generation after segregation. For CRISPR/Cas, it is also possible to deliver the CRISPR and the Cas as mRNA and guide RNA, respectively (Zhang et al., 2016) or to deliver a pre-assembled ribonucleoprotein (RNP-complex) (Woo et al., 2015). RNA and RNP delivery completely exclude any introduction and integration of foreign DNA into the plant.

The lack of foreign DNA in the NBT mutated plants complicates the traceability, which is required when NBT mutations are regulated as GMOs. Traditional GM plants normally holds a large piece of foreign DNA inserted randomly in the plant genome. Today, GM plants are detected by standard or real-time PCR that, depending on the primers used, can identify specific gene elements, gene constructs, and transformation events present in the plants. Knowledge about the sequences to be identified is a prerequisite for design of the primers. With respect to traceability of NBT mutations, it will not be possible to separate mutations resulting from SDN1, SDN2, or ODM from mutations induced spontaneously or by conventional mutagenesis even if information about the gene sequences is available. The same goes for base editing. This will complicate the control of crops imported from countries that do not regulate NBT mutated crops.

### Off-Target Mutations Induced by NBT Tools

An often-mentioned concern about the ODM and SDN-tools is if mutations are generated at places in the genome where the tools were not intended to mutate. These so-called off-target mutations occur when the tool is capable of binding and inducing DSBs within sequences similar to the sequence which the tool was designed for (off-target sequences). Both ODM and all the SDN-tools may induce off-target mutations but the frequency is higher with the CRISPR/Cas9 tool (Zischewski et al., 2017). The reason for this is a less specific binding capacity. For CRISPR/Cas9, the recognition sequence is a 20-nucleotide sequence complementary to a 20-nucleotide genomic sequence located where the mutation is intended. Although a 20-nucleotide gRNA recognition sequence is long enough to occur only once in the vast majority of plant genomes, the specific binding is highest for the 8 to 12 nucleotides of the gRNA following the PAM sequence. This means that the gRNA can bind to sequences where there are mismatches between the gRNA and the plant DNA in the last 8 to 12 nucleotides (Hsu et al., 2013; Pattanayak et al., 2013; Cho et al., 2014).

It is difficult to make a general estimate of the off-target mutation frequency induced by the CRISPR/Cas9 tool in plants. In most of the CRISPR/Cas9 mutated crops developed, no analyzes have been performed for off-targets. Out of 1328 studies using CRISPR/Cas, TALENs, base editing, ZFN, and ODM, 252 of them investigated off-target mutations. In around 3% of the analyzed potential of-target sites, unintended mutations were detected (Modrzejewski et al., 2019).

Examples of studies where the CRISPR/Cas9 off-target mutation frequencies have been investigated by PCR amplification of the off-target sequences, restriction fragment analysis and/or sequencing and where off-target mutations have been identified are shown in **Table 2**. The studies included show off-target mutation frequencies at these sites ranging between 0% and 67.5%, depending on the targeted sequence and show that off-target mutations are often induced when there are mismatches at positions 8 to 20 from the PAM sequence.

**Table 2:** Examples of studies where off-target mutations induced by CRISPR/Cas9 were identified by PCR/RE and/or sequencing.

Reference	Species	Delivery method	Potential off-target sites	Homologous genes
(Xie and Yang, 2013)	Rice	Stabile CRISPR/Cas9	Off-target mutation in one of three potential off-target sites containing a mismatch at position 11 and 15 from the PAM with a mutation frequency of 1.6%	
(Endo et al., 2015)	Rice	Stabile CRISPR/Cas9		For one gRNA, off-target mutations were investigated in 3 homologous genes.
				One gene contained mismatches at positions 7, 16, and 18 from the PAM and showed no off-target mutations in 31 plants.
				Another gene contained a mismatch at position 18 from the PAM and showed off-target mutations in 19 out of 31 (61.3%) plants.
				The third gene contained mismatches at positions 10 and 16 from the PAM and showed off-target mutations in 17 (54.8%) out of 31 plants
(Zhang et al., 2016)	Wheat	Stable CRISPR/Cas9 or Transient DNA CRISPR/Cas9 or Transient RNA CRISPR/Cas9	For one gRNA the software predicted eight potential off-target sites containing different mismatches at positions within the 20 to 6 bp from the PAM. No off-target mutations were identified in a total of 67 regenerated mutants generated by either delivery method.	For one gRNA, off-target mutations were investigated in one homeologues gene with a mismatch at position 9 from the PAM. For stabile delivery with DNA, transient delivery with DNA. and transient delivery with RNA, off target mutations were identified in 2.0%, 2.3% and 0.4% of the regenerated mutants, respectively.
			For another gRNA the software predicted 24 potential off-target sites containing different mismatches at positions within 20 to 1 bp from the PAM. No off-target mutations were identified in a total of 101 regenerated mutants generated by transient DNA delivery.	
(Li et al., 2016)	Rice	Stable CRISPR/Cas9	For four different gRNAs, two potential off-target sites were investigated. Within these eight sites, off-target mutations were identified at three sites containing mismatches at position 13, 14 + 16–20, and 8 from the PAM with mutation frequencies of 67.5%, 2.5% and 47.5%, respectively.	

Off-target mutation frequencies can also be estimated by whole-genome sequencing (WGS). In order to get maximum information from this method, the appropriate controls need to be included, revealing also the mutagenesis effect of tissue culture and CRISPR/Cas.

In a recent study in rice, such an approach was used to distinguish pre-existing mutations, spontaneous mutations, and mutations caused by tissue culture and *Agrobacterium*-mediated transformation from off-target mutations (Tang et al., 2018). No off-target mutations were found in plants edited by 11 out of 12 different Cas9-gRNA. The off-target sequences of the one Cas9-gRNA where off-target mutations were found also contained mismatches at positions 1 to 8 from the PAM sequence. This indicates that in order to avoid off-target mutations there should be at least two mismatches at positions 1 to 8 from the PAM sequence between the target sequence and any potential off-target sequences. However, the most surprising result of the study was that the highest frequency of mutations in the edited rice plants were created by the tissue culture process which caused 102 to 248 single nucleotide variations and 32 to 83 indels per mutated plant.

Similarly a study in cotton demonstrated that the most variations following Cas9-editing are due either to somaclonal variation or/and pre-existing/inherent variation from maternal plants, but not off-target effects (Li et al., 2019).

Despite the off-target mutations caused by NBT mutagenesis, non-planned mutations are still generated at much lower frequencies by the SDN-tools than by conventional mutation breeding. Here thousands of mutations may co-occur in every plant of a mutant TILLING population screened for a desired mutation (e.g. (Krasileva et al., 2017; Szarejko et al., 2017).

#### Precautions Against CRISPR/Cas Off-Target Mutations

Regardless of the very low SDN-based off-target mutation rates when compared to conventional mutagenesis, various strategies have been developed to further avoid or minimize off-target mutations by CRISPR/Cas. For plant species where the whole genome sequence is available, the main strategy is to design a very specific guide RNA sequence and to check for the presence of off-target sequences in the genome to which the guide RNA sequence could bind more non-specifically. Different software platforms have been developed to design guide RNA sequences which will very specifically bind to the sequence where the desired mutation is intended.

For plants where the genome has not yet been fully sequenced various strategies can be used to reduce the risk of off-target mutations. The CRISPR/Cas9 specificity can be improved by increasing the number of nucleotides required to recognize corresponding nucleotides in the plant genome. This can be done using the Cas9 nickase or Cas9 FokI fusion proteins strategies, which both greatly reduce the number of possible off-target sequences [reviewed by (Bortesi and Fischer, 2015)]. Another strategy is to use the newly developed spCas9-HF or the Cas12a nuclease, both possessing higher specificity (Kleinstiver et al., 2016a; Strohkendl et al., 2018). The delivery method used for the CRISPR/Cas9 tool to the cells also greatly influences the frequency of off-target mutations. Studies have shown that delivery of the CRISPR/Cas9 tool as RNP complexes reduce the number of off-target mutations since RNP complexes degrade much faster in the cell than DNA constructs (Kim et al., 2014; Liang et al., 2017).

RNP delivery seems to be one of the most promising tools for reducing off-targets. The RNP delivery is, however, currently only possible by protoplast transfection or by particle bombardment (Woo et al., 2015; Liang et al., 2017). Currently, this puts some limitations on a broad use of RNP as regeneration of plants from protoplasts is only possible from rather few plant species and highly efficient protocols for particle bombardment and plant regeneration is limited to a few species. Future developments might increase the number of plant species where RNP delivery is possible and make RNPs the preferred way of CRISPR/Cas delivery. Moreover, the number of plant species with fully sequenced genomes is constantly increasing and expands the number of plant species where maximum specific guide RNA sequences can be designed.

## Discussion and Conclusions

The status of new breeding technologies (in particular SDN1 tools) with respect to the EU GMO legislation is important for the possibilities to exploit the potentials of the technologies in future European plant breeding. The ruling of the EU Court of Justice in July 2018 stating that organisms obtained by the new mutation techniques are not exempt from the legislation on the deliberate release of GMOs, makes it difficult, if not impossible, for plant breeders to make use of the new techniques, due to heavy costs associated with the approval of GM varieties (Eriksson et al., 2018). Realistically, only big companies can afford the costs and, hence, only these companies can probably make commercial use of the new genome editing technologies. On short terms, the influence on the European plant breeding industry, in particular small and medium size enterprises (SMEs), might not be strong although quite a number of SMEs may have stopped their own SDN projects after this ruling. However, in the longer perspective, this industry will probably stand weak in the competition with countries outside EU, like the US where USDA APHIS has formulated a policy in which crops that contain single nucleotide changes or deletions of any size would no longer be a regulated article, and Argentina and Brazil which have installed a process that results in that certain SDN plants are not subject to the provisions of their GMO legislation.

Paradoxically, mutant plants can now be achieved with mutagenesis methods exempted from the GM legislation just as fast as with the SDN1 techniques. Hence, with respect to targeting and speed a convergence between conventional mutagenesis and the SDN1 techniques has occurred. The two major differences are the precision and the number of off-target mutations, both of which favour the SDN1 methods.

With the current status of the EU legislation of plants with NBT mutations, reluctance by private industry to embark on projects implementing these techniques in generation of new varieties will probably persist. Already now, Europe falls behind with respect to patenting within the area of CRISPR-based plant biotechnology (Martin-Laffon et al., 2019). However, the techniques could still be used with success in research projects that address the molecular genetics of crop traits. Hand-in-hand with the use of classical mutation techniques through screening of TILLING populations NBT mutations could be utilized indirectly, although not optimally, in the modulation of crop traits. First, the precision and specificity of the NBT mutations could be used to clearly define strong mutation targets, without the genetic noise that would be present in classical mutation strategies. Subsequently, the defined efficient mutations could be re-constructed/re-gained by the use of classical mutation techniques. This is a cumbersome process, but still applicable, in particular due to the development of efficient methods to build mutant (TILLING) populations and efficient methods to screen them for mutations of specific target genes (Jankowicz-Cieslak et al., 2017).

The development of crop varieties usually takes many years and, thus, the effects on the market and in agriculture will have an equivalent lag. For proper exploitation, it is therefore important that implementation of the new precision breeding techniques is started now, maybe in strategies in combination with TILLING approaches as outlined above. The fear, however, could be that the decision by the EU court will still make the industry reluctant to go into research and development directed toward the use of the new techniques. This would only aggravate the long term weakening of the European breeding industry in the global competition.

For some crops and traits, in particular ornamentals and garden/vegetable seeds, new cultivars have a short developmental horizon, e.g., when it comes to developing new flower colours. Since the market of these is global, the impact of the new techniques on Europe can come on rather short terms, if restrictions on their use, as expected, will be limited in Asia, South America, Canada, and the US. This raises the issues of detection of genetic modifications introduced by the new techniques. In Europe, they are regulated according to the current EU GMO legislation and, hence, subject to strict approval, but it is close to impossible to make unambiguous tests for the introduced mutations, since there are no marks distinguishing them from natural mutations/variants. This situation will get even worse on longer terms, when varieties of major European crops with new NBT mutation induced traits can enter the European market from the surrounding world and be crossed with locally developed varieties. From the regulatory aspect, this situation will not be sustainable and thus unacceptable for the European plant breeding industry. Long-term stability in this area calls for clarifications at the political level of EU legislation.

## Author Contributions

IH, PG and HB contributed equally to the manuscript.

## Funding

The study was supported by Innovation Fund Denmark, grant 8055-00038B, ReTraQue.

## Conflict of Interest

The authors declare that the research was conducted in the absence of any commercial or financial relationships that could be construed as a potential conflict of interest.
